# Supplementary material for: Are carbon emissions trading and green financial instruments synergistic? -Comprehensive quantitative research based on content analysis
Source: PLoS One. 2024 Mar 7;19(3):e0298601. doi: 10.1371/journal.pone.0298601 (PMC10919601; doi:10.1371/journal.pone.0298601)
Supplement: S1 Data — (ZIP) [file pone.0298601.s001.zip › Data/地级市Z_F工作报告.zip!/╣π╬≈/║╙│╪╩╨/└┤╫╘╣1⁄2╓┌║┼í╛┬φ┐╦╩2╛▌═°í┐_2017.docx]

各位代表：

现在，我代表市人民政府向大会作政府工作报告，请予审议，并请政协委员和列席会议的同志提出意见。

一、2016年工作回顾

过去一年，面对经济下行压力不断加大、多重困难矛盾相互交织、产业转型升级任务异常繁重的严峻形势，在自治区党委、政府和市委的正确领导下，在市人大、市政协的监督支持下，我们全面贯彻党的十八大和十八届三中、四中、五中、六中全会精神，深入贯彻习近平总书记系列重要讲话精神和治国理政新理念新思想新战略，牢固树立和贯彻落实新发展理念，全力推动经济、政治、文化、社会和生态文明建设，全市呈现经济稳中有进、民生持续改善、社会和谐稳定的良好局面，实现了“十三五”良好开局。初步预计，全市完成地区生产总值650亿元，增长5%；规模以上工业增加值135亿元，增长3%；财政收入62.2亿元，增长10.9%；社会消费品零售总额268亿元，增长10.1%；固定资产投资416亿元，增长5%；全体居民人均可支配收入13175元，增长9.5%，其中，城镇居民人均可支配收入23660元，增长6.4%，农村居民人均可支配收入7509元，增长8.4%。

一年来，我们主要做了以下工作：

（一）实施精准扶贫，脱贫攻坚实现良好开局。举全市之力，全面实施“七个一批”和19个配套方案，115个贫困村、20万贫困人口有望实现脱贫摘帽。易地扶贫搬迁、产业脱贫、旅游扶贫、小额信贷、“空店”精准扶贫等工作经验在全区推广。产业扶贫方面，坚持“长中短”结合，大力发展核桃、桑蚕、特色水果等十大扶贫产业，“一县一品牌、一乡一特色、一村一品种、一户一项目”建设扎实推进。新种植核桃25万亩，总面积达241万亩，2012年种植的核桃效益初显，实现产量2000吨、产值8000万元。易地扶贫搬迁方面，91个移民安置项目（点）全部开工建设，完成投资34.9亿元，建成住房10413套，搬迁入住38907人，入住率50%，综合进度排在全区前列。基础设施建设方面，脱贫村路、水、电、房、网等基础设施建设大为改善，全部达到“十一有”标准，其中建设屯级道路3146条5448公里，对19.9万农村群众饮水进行巩固提升，推进危房改造2.8万户。教育扶智方面，发放各级各类学生资助款3.8亿元，受助学生25.9万人次。生态补偿方面，管护生态公益林1951万亩，落实生态补偿资金3.3亿元，争取获得4830名生态护林员指标。社保兜底方面，推进农村低保与扶贫政策有效衔接，36.5万贫困群众纳入低保，重合率达85%。4.58万名干部与17.6万户贫困家庭结成帮扶对子，精准帮扶措施有效落地。医疗救助方面，对因病致贫、因病返贫的农村贫困人口，纳入重特大疾病救助范围，48.6万人享受新农合缴费补助，补助金额2739万元。资金投入方面，全市整合筹措财政资金60.7亿元投入脱贫攻坚，比上年翻一番。发放扶贫小额贷款42亿元，覆盖贫困户90120户，占预脱贫户的86.2%。扶贫协作方面，主动与深圳市对接，编制完成对口帮扶协作纲要，落实年度帮扶资金7000万元。

（二）全面落实稳增长措施，经济保持平稳健康发展。研究出台稳增长工作实施意见，提出8方面48条稳增长措施，全力以赴稳投资、稳工业、稳农业、稳消费、稳进出口、稳财政，经济运行呈现“总体平稳、稳中提质、稳中有进”的良好态势。

扩大投资上项目。健全完善重大项目协调推进机制，开展项目建设攻坚推进“百日行动”，着力突破项目审批、征地拆迁、融资等瓶颈制约。加快推进自治区和市级层面重大项目建设，新开工项目89个，竣工项目40个，完成投资217亿元，占年度计划的126%。抓好具有带动性、示范性的“十大城建”“十大工业”“十大农业”项目建设。加快中央预算内投资、左右江革命老区三年行动计划和西江基础设施建设大会战项目建设。加大项目策划力度，市本级安排前期项目经费1740万元，策划包装91个重点项目。全市重大建设项目库在库项目2414个，总投资5843亿元。

转型升级稳工业。全力支持重点产业、重点园区、重点企业，加快推进重点工业项目建设，都安西江鱼峰公司新型干法水泥、南方公司锑银多金属回收、润达制药搬迁扩能、金兴公司精细化工、五和博澳桑枝综合开发等一批采用新技术新工艺的重大项目竣工投产。工业固废综合利用率5年提高19.3个百分点，成为全国首批工业资源综合利用示范基地。深入实施服务企业大行动，着力解决制约企业发展的突出问题，促进企业稳产复产。大任产业园建设实现新突破，园区路网、供水、供电等基础设施基本完善，首批入园企业实现竣工试产。全市11个工业园区（集中区）完成总产值152.1亿元、增加值39.3亿元。优质饮用水产业保持强势增长势头，产值增长20%。质量强市显著加强，5家企业荣获广西名牌产品称号，1家企业荣获自治区服务业品牌称号。

培育特色强农业。现代特色农业示范区建设富有成效，创建各级示范区108个，辐射带动5万农户。优势种植业巩固提升，完成“双高”基地建设2.5万亩、糖料蔗种植85.4万亩，蚕桑种植82.2万亩，鲜茧产量11.5万吨，桑园面积、鲜茧产量连续11年位居全区第一。特色养殖业发展迅速，牛羊饲养量、出栏量和内陆大水面渔业养殖面积均排全区第一。特色农业品牌开发进一步提升，打造富硒农产品品牌16个、基地18个、面积2.85万亩。新型农业经营主体不断培育壮大，农民合作社发展到2607家，家庭农场发展到507家。农业、林业、水产畜牧业固定资产投资均保持两位数增长。全面完成城镇周边永久基本农田划定，耕地连续17年实现占补平衡。新增、恢复和改善灌溉面积37.5万亩，农作物耕种收综合机械化率达46%。

发展三产促消费。以旅游业为主导的现代服务业蓬勃发展，城市形象和旅游品牌进一步提升，获评中国首个地级“世界长寿市”。巴马长寿养生国际旅游区建设加快推进，实施基础设施建设项目138个，完成投资10.8亿元；核心区赐福湖小镇等5个重点项目完成投资4.78亿元，占年度任务的227%。《巴马长寿养生国际旅游区基础设施建设三年行动计划（2017—2019年）》获自治区批复并启动实施，涉及我市项目总投资22.7亿元。百里龙江民族文化生态旅游带建设步伐加快。国家公园（风景区）、4A级景区分别增至10个、12个。宜州市、巴马瑶族自治县被评为广西特色旅游名县。新建50个旅游扶贫示范点，带动1.7万贫困人口脱贫，乡村旅游工作走在全区前列。全市接待游客2164万人次，增长16.9%，旅游总消费233亿元，增长28.1%。狠抓服务业重点项目建设，创建诚信企业取得突破，10家企业被评为广西商务诚信示范单位。加快发展电子商务，成立河池电商协会，创建市本级电子商务创业园，电商企业发展到83家。39家企业在全国性股权交易市场挂牌。金融实现较快增长，全市各项存款余额1008亿元，增长13.6%；各项贷款余额576亿元，增长12.8%。

开源节流聚财力。深挖增收潜力，财政收入突破60亿元，非税收入比重逐步下降，财政收入质量进一步提高。做好政府债务置换工作，市本级置换债券26.2亿元，获新增债券3.9亿元，有效缓解资金支出压力。财政支出完成290.7亿元，完成进度96.7%，增长11.7%。支出结构明显优化，86%的公共财政预算支出用于民生事业，“三公”经费零增长。东兰县强化财税监管，财政收入提前超额完成全年预期目标任务，获得自治区政府激励表扬。

（三）大力改善基础设施，城乡建设步伐日益加快。为尽快改变河池基础设施落后面貌，谋划实施总投资842亿元的交通、水利基础设施建设大会战。交通基础设施建设大会战完成投资61.6亿元，占年度任务的113%。河池至百色高速公路建设加快推进，贵阳至南宁高速铁路、贺州至巴马高速公路（都安至巴马段）开工建设。建成凤山至凌云逻楼、天峨至凤山（凤山段）、巴马至平果、大任产业园入园等4条二级公路136公里，开工建设南丹吾隘至东兰、天峨至凤山（天峨段）、大化县城绕城公路3条二级公路，罗城至融水洞头、三岔经洛东至宜州、东兰泗孟至武篆等一批二级公路建设稳步实施。新开通西安—桂林—河池航线。水利基础设施建设大会战有序推进，重点水源、农村水利、防洪减灾等三大工程有效实施，新开工重大水利工程项目12个，完成水利固定资产投资15.8亿元。桂西北贫困地区（河池）特色农业节水灌溉项目、桂西北扶贫治旱红水河灌区工程2个重大项目纳入国家规划。新型城镇化步伐加快，宜州市撤市设区和市政府驻地迁移获国务院批复同意，启动《河池市市域城镇规划体系》《宜州新区控制性详细规划》等5个专项规划编制。集中力量完善市中心城区基础设施，老虎山片区棚户区改造等26个重点项目积极推进，辉乐桥、河池市体育场及地下停车场等项目开工建设，百旺路等一批路网工程竣工使用。河池市水电园林广场荣获2016至2017年度第一批中国建设工程鲁班奖。宜州、环江、东兰3个县（市）入选全区新型城镇化示范县。巩固“城乡新貌新风工程”，开工建设城乡新貌新风实体项目362项，完成投资46.8亿元。

（四）坚持绿色发展，生态文明建设进一步加强。被列为全国土壤污染综合防治先行区，争取国家专项资金3.6亿元，并率先启动实施。强化水污染防治，推进龙岩滩水库生态环境保护项目建设，主要河流水质均达到国家标准，水功能区平均水质达标率100%。全面完成黄标车、老旧车淘汰任务，市中心城区空气环境质量优良率达95%。加大植树造林力度，完成造林37.4万亩，封山育林1.5万亩，退耕还林13.7万亩，石漠化综合治理4587亩，森林覆盖率68.8%。罗城、环江两县纳入国家重点生态功能区。生态乡村建设扎实推进，绿化示范村屯560个，绿化一般村屯9514个，新增绿化面积1.1万亩，村屯绿化规模及竣工率均排全区前列。

（五）深化改革扩大开放，发展活力得到有效激发。推进供给侧结构性改革，“三去一降一补”初见成效，全面落实降成本41条措施，累计为企业降低成本5亿元，其中，通过电力直接交易等方式，为企业降低成本2.5亿元；全面推开“营改增”，免征税款961.6万元。化解煤炭过剩产能123万吨，占全区总任务的50.7%。实施棚户区改造货币化安置，鼓励补助新市民购房，商品房待售面积减少7.4万平方米。加强债务管理，强化风险防控。加大力度补短板，教育、卫生等领域投资均实现两位数以上增长。深化行政审批制度改革，大力推行“两个清单”，取消下放行政审批事项134项。加快推进国资国企改革，推动经营性国有资产统一管理，妥善处理企业改制遗留问题，企业重组工作取得新成效，市属国有企业实现融资41.5亿元，发行企业债券15亿元。推进商事制度改革，新增企业3896户，增长31%，注册资金157亿元，增长26%。完成环江、南丹两县国有林场改革试点工作，城市公立医院改革正式启动，科技、教育、供销社等领域改革取得实效。科技创新能力日益增强，申请专利1501件，增长46.3%；获得专利授权504件，增长68%。公务用车改革工作扎实推进，市本级核减取消公务用车399辆。全面推行不动产统一登记。出台招商引资系列配套政策，全市招商引资内资到位300.8亿元，增长11.7%，全口径实际利用外资1.3亿美元，增长8.2%。

（六）保障和改善民生，群众生活水平不断提高。为民办好十件实事提升了人民群众的获得感和幸福感。教育教学指标全面完成，学前教育三年毛入学率达78.8%，义务教育巩固率达92%、高中阶段毛入学率达84%。大力促进就业创业，新增城镇就业人口1.7万人，新增农村劳动力转移就业5.8万人次，城镇登记失业率2.86%。城镇职工“五险”参保人数113.2万人，新农合基本实现全覆盖，参合率达99.3%。建成保障性住房和棚户区改造4256套，分配入住4439套。人口和计划生育工作扎实推进，“全面两孩”政策顺利实施。开工建设136个村级公共服务中心，铜鼓文化（河池）生态保护实验区总体规划通过文化部评审。金城江区红军标语楼、宜州市会仙山摩崖石刻和环江县凤腾山古墓群3个项目列入国家“十三五”文物保护利用规划。全民健身活动蓬勃开展，竞技体育成绩喜人，运动员彭秋萍在里约残奥会勇夺2金1银。落实岩滩水电站水库移民遗留问题处理规划，实施项目342个，完成投资2.2亿元，库区移民生产生活条件得到改善。投入资金3137万元治理地质灾害点29处，保障7000多人的生命财产安全。民族政策全面贯彻落实，成功举办巴马瑶族自治县成立60周年县庆活动。发挥价格调节基金作用，实施“菜篮子”工程，物价水平保持稳定，居民消费价格指数控制在1.5%左右。食品药品监管不断强化，日常监管实现全覆盖。全市安全生产形势持续稳定。平安河池、法治河池建设成效明显，群众安全感保持全区前列。国防动员和后备力量建设不断加强，双拥共建力度加大，东兰县被评为全国双拥模范城，河池市、天峨县被评为全区双拥模范城。

（七）切实加强自身建设，依法行政能力不断提升。严格落实中央八项规定精神和国务院“约法三章”。扎实开展“两学一做”学习教育，认真学习贯彻《中国共产党廉洁自律准则》《中国共产党纪律处分条例》《关于新形势下党内政治生活的若干准则》《中国共产党党内监督条例》。制定出台法治政府建设实施方案，着力提高政府依法履职、依法行政能力。认真执行市人大及其常委会的决定决议，自觉接受市人大及其常委会的法律监督、工作监督和市政协的民主监督，认真办理人大代表议案、建议和政协委员提案，办复率100%。进一步完善政府议事决策规则，实行重大行政决策专家咨询论证，重大决策程序和机制进一步完善。扎实开展地方政府立法相关工作，起草完成《河池市非物质文化遗产保护条例（草案）》。深化政务公开，网络问政制度化、规范化、常态化运行。加大行政监察和审计监督力度，推进党风廉政建设和反腐败工作，深入查处发生在群众身边的“四风”和腐败问题，严肃查处了一批违纪案件，群众对反腐败成效满意度进一步提升。

统计、司法行政、税务、外事、宗教、侨务、机关事务、消防、保密、检验检疫、档案、地方志、通信、海事、气象、地震、水文等工作迈出新步伐。老干部、妇女儿童、老龄、残疾人保障等事业取得新进展。工会、共青团、妇联等群团组织在经济社会发展中发挥了重要作用。驻河池中直、区直各单位支持地方发展和自身业绩取得新成效。

各位代表！刚刚过去的2016年，我们凝心聚力、砥砺奋进，顶住了压力，经受住了考验，发展质量效益不断提升，为我市打赢脱贫攻坚战、全面建成小康社会奠定了坚实基础。这是市委统揽全局、科学谋划的结果，是市人大、市政协有效监督、鼎力支持的结果，是全市广大干部群众团结一心、合力攻坚的结果，是离退休老干部鼓励支持、建言献策的结果，是驻河池人民解放军、武警部队和社会各界广泛参与、大力支持的结果。在此，我谨代表市人民政府，向全市各族人民，向人大代表、政协委员，向各民主党派、工商联、无党派人士、各人民团体，向驻河池人民解放军、武警部队官兵，向所有关心支持河池发展的社会各界人士，表示崇高的敬意和衷心的感谢！

在总结成绩的同时，我们也清醒地认识到，受历史及自然地理条件等因素制约，河池仍然是一个欠发达后发展地区，还面临着一些困难和问题：经济转型难，重大项目不多，新的增长动能尚在培育当中，经济下行压力依然较大；贫困人口多，贫困面大，贫困程度深，脱贫攻坚任务艰巨；政府效能、服务水平还不够高，部分干部干事创业、克难担当的精神还不够，发展环境还需进一步优化，等等。我们必须直面这些问题，切实增强忧患意识、责任意识、担当意识，努力在破除瓶颈、补齐短板上取得明显进展，把政府各项工作做得更好，不辜负全市人民的期望。

二、2017年工作安排

2017年是实施“十三五”规划的重要一年，也是供给侧结构性改革的深化之年，更是打好脱贫攻坚战的重要之年。当前，国家深入实施“一带一路”、左右江革命老区振兴规划等重大战略部署，加大对革命老区、贫困地区、少数民族地区的扶持力度；国家深入推进“三去一降一补”、推进农业供给侧结构性改革、着力振兴实体经济、促进房地产市场平稳健康发展、深化国企国资改革等一系列促改革、稳增长政策措施，加快新旧动力转换接续；这些为我们拓展发展新空间、培育发展新动能带来重大政策利好。同时，河池市行政区划调整获国务院批复，我市迎来了重大历史性发展机遇；有色金属价格回升，出城入园企业陆续投产，企业发展向好因素增多；而且我市正在实施脱贫攻坚战，这既是最大的政治责任，更是最大的民生工程和最大的发展机遇。我们一定要坚定信心，振奋精神，鼓足干劲，着力破解发展难题、补齐发展短板、厚植发展优势，开创新常态下河池改革发展新局面！

今年政府工作的总体要求是：深入学习贯彻党的十八大和十八届三中、四中、五中、六中全会精神以及中央、全区经济工作会议精神，深入贯彻习近平总书记系列重要讲话精神和治国理政新理念新思想新战略，统筹推进“五位一体”总体布局，协调推进“四个全面”战略布局，紧紧围绕自治区第十一次党代会提出的营造“三大生态”、实现“两个建成”战略部署和市第四次党代会提出的“1234”总体工作思路，以新发展理念引领经济发展新常态，以开展“六大行动”推进年为抓手，全力稳增长、促改革、调结构、惠民生、防风险，有序稳妥推进行政区划调整，促进全市经济平稳健康发展、社会和谐稳定，以优异成绩迎接党的十九大胜利召开！

全市经济社会发展的预期目标是：地区生产总值增长6%，规模以上工业总产值增长10%，规模以上工业增加值增长5%，固定资产投资增长10%，财政收入增长6%，社会消费品零售总额增长9.4%，全体居民人均可支配收入增长7.7%，居民消费价格涨幅控制在3.5%，城镇登记失业率控制在4.5%以内，常住人口城镇化率38%，节能减排控制在自治区下达目标内。

这些目标，是立足实际和可能，衔接全面建成小康社会和“十三五”规划各项指标，经过反复比较、综合权衡后确定的，既保证经济运行在合理区间，又保障供给侧结构性改革、民生改善、财政增长，为调结构、防风险，增强社会和市场信心创造条件。我们必须全面贯彻、自觉践行新发展理念，开展营造“三大生态”（风清气正的政治生态，团结和谐的社会生态，山清水秀的自然生态），实施“三大攻坚”（精准脱贫、基础设施建设、产业转型升级）六大行动推进年活动，优化发展路径，拓宽发展空间，增强发展动力，在全面建成小康社会新的长征路上迈得更稳、走得更扎实。

围绕实现上述目标，重点做好八个方面的工作：

（一）实施精准脱贫攻坚行动，坚决完成脱贫摘帽任务。坚持精准扶贫、精准脱贫基本方略，大力实施脱贫攻坚“七个一批”，实现金城江区、南丹县2个县（区）摘帽、182个贫困村出列、19.6万人脱贫。

抓好产业扶贫。产业发展是脱贫攻坚的关键。组织实施“十三五”产业精准扶贫发展规划，按照“长中短”相结合，继续抓好核桃、糖料蔗、桑蚕、蔬菜、食用菌、经济林、特色水果、草食动物生态养殖、水产养殖、特色畜禽养殖等十大扶贫主导产业，实现扶贫产业对贫困户全覆盖，通过产业扶贫最终实现稳定脱贫。采取有力措施加强核桃管护，确保成活率和挂果率，新增核桃种植10.5万亩、补植14万亩。扎实开展“产业富民”专项活动，加大产业项目资金投入，确保项目到村到户、资金到村到户到人。推广“空店”精准扶贫模式，大力发展电子商务、农超对接等现代流通方式，拓宽贫困村特色农产品销售渠道，增加贫困群众收入。大力扶持村级集体经济发展，确保每个贫困村集体经济收入到2020年达到5万元以上。

抓好易地扶贫搬迁。这是脱贫攻坚最难啃的“硬骨头”。采取过硬得力的措施，推动易地扶贫搬迁实现“两个全部”目标，即“十三五”易地扶贫搬迁项目在2017年全部开工建设、2019年前全部建成入住。其中，今年完成投资72.7亿元，搬迁27381户114068人。全面把握政策，严格执行建档立卡贫困人口住房建设面积标准，严禁挪用其他专项资金或举债扩大住房建设。强化搬迁群众后续发展能力建设，同步建设创业园，引进企业吸纳就业，扶持群众发展产业、自主创业、确保“搬得出、稳得住、可发展、能致富”，最终实现贫困群众挪穷窝。

抓好基础设施建设。这是脱贫攻坚的基石。认真策划脱贫攻坚项目，建立县、乡、村项目库，通过储备项目争取上级资金支持。对照贫困村脱贫“十一有”标准，集中财力抓好182个贫困村路、水、电、房、网等基础设施建设。投入1.2亿元，硬化改造54个贫困村通村道路180公里，实现今年摘帽贫困村通村道路全部硬化；投入4.7亿元，修建589条20户以上自然屯屯级道路1488公里；建成91个贫困村村级公共服务中心；实现44个行政村通宽带网络；解决1.2万贫困人口的饮水安全问题；保障630户贫困人口安全用电。加快资金拨付，加快项目建设，确保项目发挥效益，不断改善贫困地区生产生活条件。

抓好教育扶智扶贫。教育是改变河池、拔掉穷根的根本。精准落实教育扶贫优惠政策，实现从学前教育到高等教育各阶段学生资助全覆盖，资助贫困家庭学生28.6万人次以上，确保每个学生不因贫失学、每一个家庭不因学返贫。继续实施农村义务教育学生营养改善计划，让23万名学生吃上营养餐。加强农村劳动者尤其是贫困人口职业技能培训，完成农民科技培训20万人次，新型职业农民培训2000人。教育引导贫困群众增强脱贫意识，树立自立自强、勤劳致富、脱贫光荣的思想，充分激发群众脱贫内生动力。

抓好金融扶贫工作。新增扶贫小额信贷资金5亿元以上，累计贷款余额达45亿元以上，发放贴息1.5亿元，引导金融资金投入贫困地区和贫困农户，缓解贫困户贷款难、贷款贵等问题，支持发展扶贫产业，加快脱贫增收步伐。做好保险助推脱贫攻坚工作，实施贫困人口意外伤害保障、家庭财产安全、政策性农业保险等项目，实现贫困地区保险服务到村到户到人。

抓好脱贫攻坚机制落实。强化党政主要领导负总责的脱贫攻坚责任制，层层压实脱贫攻坚责任。深入开展定点扶贫，深化干部结对帮扶，带着责任和感情进一步落实好精准帮扶措施，帮助贫困群众出思路、谋计策、做实事。高标准严要求做好“一户一册一卡”的填写发放工作。从始至终重视抓好脱贫攻坚档案材料的收集整理，按照贫困县“九有一低于”、贫困村“十一有一低于”、贫困户“八有一超”脱贫摘帽核验标准，对各项指标涉及的文字、图片等佐证材料进行全面收集和规范整理，让帮扶干部和贫困群众做到心中有数，进一步提高精准识别准确率、精准退出准确率和群众满意度，经得起各方的考核评估。关心爱护、支持帮助乡镇干部、第一书记和工作队员，强化教育、培训、管理、考核和保障，为他们开展工作创造条件，充分调动基层干部的积极性。用好督促检查和考核评估结果，对任务完成不好的，该约谈的约谈，该通报的通报；对搞形式主义、弄虚作假和造成不良后果的，严肃问责。拓宽与深圳市的扶贫协作，坚持专项扶贫、行业扶贫和社会扶贫联动，积极引导社会力量参与帮扶，形成全社会共同参与扶贫的强大合力。同时，统筹抓好旅游扶贫、生态补偿、医疗救助、转移就业、社保兜底等工作。

（二）实施产业转型升级攻坚行动，推动工业提质发展。以提高工业发展质量和效益为中心，实施创新驱动发展，加快传统产业转型升级，振兴实体经济。

加强园区平台建设。加快推进市工业园区、河池•南丹工业园区、河池•东莞工业加工二区（广西宜州经济开发区）、河池•环江工业园区、河池•都安临港工业区等市级工业园区建设，统筹推进市县联办工业园区和县级园区建设，增强园区承载能力。每个市级工业园区力争策划一个亿元以上项目、跟踪落地一个亿元以上项目、开工建设一个亿元以上项目、竣工投产一个亿元以上项目、实施一个亿元以上转型升级项目、实施一个亿元以上新兴产业项目。突出抓好大任产业园建设，竣工园区路网、污水处理厂、送变电工程等基础设施项目，推动城区及周边企业搬迁入园，力争新增4家以上企业实现竣工投产，实现总产值10亿元以上。全市工业园区实现总产值230亿元以上，完成项目投资15亿元以上，基础设施投入3.5亿元以上。

改造升级传统产业。采用新材料、新技术、新工艺，推动有色金属、茧丝绸、制糖等传统产业改造提升。推动有色金属产业“二次创业”，加快建设生态环保型有色金属产业示范基地，推动矿产资源优先配置给本地强优企业。建立南方公司铅锌锑工程技术研究中心、广西有色金属新材料研究中心等科技创新平台，推动关键共性技术攻关和成果转化应用。重点推进南方公司次氧化锌、3万吨锡冶炼，鑫锋公司废旧电池回收、高峰公司巴里选矿厂技改、正华冶炼厂技改、明鑫公司工业危废无害化处理、五吉公司3万吨锑等项目建设，有色金属产业新增产值30亿元以上。提升缫丝加工水平，扩大坯绸产能，开工建设嘉联丝绸园、恒业公司印染项目，加快织绸技改项目，推动茧丝绸产业扩能增效，实现产值28亿元以上。推动都安永鑫公司重组，支持广维化工公司、博冠纸业公司做强糖蜜、蔗渣深加工，延长产业链，制糖业实现产值25亿元以上。

培育新的增长点。积极推动特色酒、优质饮用水、绿色长寿食品等产业规模化、品牌化、高端化发展。推进丹泉、天龙泉、德胜红兰酒业等公司扩能技改项目建设，酿酒产业实现产值35亿元以上。引进大企业大集团，整合优质饮用水企业，着力解决好饮用水品牌散、规模小、效益低等问题，不断扩大罗城九千万、环江木论思泉、巴马丽琅、凤山寿源活泉等知名品牌影响力和市场占有率，实现产值20亿元以上。开展生态原产地保护，延长特色农产品加工链条，形成一批绿色长寿食品品牌。

扶持强优企业。认真落实降成本41条措施，继续深入开展服务企业大行动，对南方公司、丹泉公司等强优企业实行“一企一策”帮扶，帮助企业解决土地、融资和降成本等问题，促进企业正常开工、高效运行、增产增效。尤其是积极巩固和争取电力直接交易量，扩大与周边省份直购电范围和规模，力争交易电量增长60%。狠抓规模以上企业培育工作，力争新增规模以上企业15家。着力推动科技创新园区、可持续发展实验区、科技创新平台、高新技术企业和科技型企业、科技企业孵化器、众创空间“六大建设”，加强品牌、产业组织和商业模式创新，推动创新驱动质量升级。培育创新型试点企业3家、高新技术企业5家，知识产权优势企业5家。

（三）大力发展现代特色农业，拓宽农民增收致富渠道。把增加绿色优质农产品供给放在突出位置，在确保粮食安全的基础上，优化农业产业结构，发展特色优势产业，加快农业发展方式转变，增强农业综合效益和竞争力。

创建现代特色农（林）业核心示范区。抓好示范区基础设施建设，拓展示范区建设规模，提升示范区科技含量，增加绿色优质农产品供给，发挥示范区辐射带动效应，新建自治区级示范区2个、市级5个、县级8个、乡级15个。

打造特色优势产业。实施科技兴农工程，加快种养殖业新品种新技术新机具推广步伐，建设重要农产品生产保护区、特色农产品优势区。桑园面积保持在82万亩，新种水果5万亩、总面积88万亩，抓好低产油茶林改造、总面积保持90万亩，建设2.5万亩“双高”基地、糖料蔗面积保持在80万亩，栽培食用菌1.7亿棒，种植蔬菜122万亩，发展牲畜养殖320万头，水产养殖8.5万吨。

培育特色农业品牌，扶持新型农业经营主体。加强农产品质量安全监管，打响“绿色长寿养生”品牌，增加绿色优质农产品供给，新增认定无公害农产品、有机食品、绿色食品、农产品地理标志产品“三品一标”农产品11个，打造富硒农产品品牌22个。加大科技助农力度，新增自治区级农业科技园区2家以上。实施“互联网+”现代农业行动，扩大特色农产品精深加工，新增农民合作社250个、家庭农场110家。

（四）推进现代服务业提质增效，培育发展新动能。坚持生产性服务业与生活性服务业、传统服务业与现代服务业并重，挖掘消费需求，培育热点消费，促进现代服务业业态多元化。

大力发展全域旅游。树立全域旅游发展的理念，用好“世界长寿市”品牌，加快构建“一区一带”旅游发展新格局，力争全年接待游客增长15%，旅游总消费增长26%。全面实施《巴马长寿养生国际旅游区基础设施建设三年行动计划》，加快推进巴马命河•水晶宫景区、百魔洞度假区、凤山世界地质公园、天峨龙滩大峡谷、东兰坡豪湖湿地公园、都安三岛湾、大化红水河百里画廊等景区景点建设。建成巴马赐福湖国际养生度假小镇项目。启动红水河全域旅游开发项目建设，加快建设百里龙江民族文化生态旅游带，重点推进金城江六甲小三峡、宜州龙洲岛生态旅游度假区、罗城剑江、环江喀斯特、南丹丹泉酒文化旅游城项目建设。以巴马为营销核心，重点推介世界寿乡养生、东巴凤长寿金三角、刘三姐民族风情、世界地质公园探险、红水河黄金水道、全国红色经典景区游6条精品旅游线路，全方位扩大河池旅游知名度和影响力。打造东兰红色文化城，筹建河池拔群干部学院。推动休闲农业和生态乡村旅游融合发展，新建旅游扶贫村28个，新增乡村旅游区14个、农家乐27家，乡村旅游消费增长25%。

积极发展大健康产业。围绕建设桂西北养生养老长寿产业示范区，以巴马为龙头，引进大企业建设养老社区、医养结合项目，打造大健康产业基地。打造长寿品牌，办好“巴马论坛”。巴马县甲篆镇、巴马镇、凤山县三门海镇、东兰县武篆镇4个镇争创全区健康养老养生小镇。

积极发展商贸物流业。规划建设一批集批发物流信息服务于一体的商贸中心、专业市场和配送中心。全力推进建材城、汽车配件城、物流仓储基地等项目建设。抓好巴马、东兰全国电子商务进农村试点工作，建立健全市县乡村四级电商服务网络。引导企业推行“线上推广交易+线下体验服务”新模式，积极开展与阿里巴巴、京东商城、苏宁易购、邮乐购、乐村淘等第三方网络交易平台企业合作，带动商贸物流业上档次、上水平。

协调发展金融等各类服务业。全力支持各级金融部门在河池设立分支机构，积极发展证券、保险、期货等各类金融业务。推进“政银企”合作，建立各类政策性基金，拓宽中小企业融资渠道，新增贷款50亿元以上。培育壮大信息科技、健康养生、会展营销等现代新兴服务业，推进交通运输、住宿餐饮、家政服务等传统产业提质升级。积极培育民族服饰、民族表演等特色文化产业，扶持壮大一批文化企业。

（五）实施基础设施建设攻坚行动，增强发展后劲。坚持把扩大有效投资作为支撑经济稳增长的有力抓手，全年完成固定资产投资460亿元左右，其中，实施自治区和市级层面统筹推进重大项目330个，完成投资260亿元以上。

推进交通基础设施建设大会战。完成投资90亿元以上。推进贵阳至南宁高速铁路项目建设。加快河池至百色高速公路、贺州至巴马高速公路（都安至巴马段）建设，开工建设融水至河池高速公路、乐业至百色高速公路（天峨段），力争开工建设南丹至天峨下老高速公路。实施国省道公路提级改造，开工建设环江至河池、宜州至龙头、罗城至环江、乐业至凤山（河池段）等项目，完成环江至东江、都安县城绕城公路、三岔经洛东至宜州、武篆至泗孟等公路建设任务。加快推进岩滩升船机改造、大化船闸改扩建前期工作以及龙滩升船机工程建设。紧盯铁路中长期规划，配合开展黔桂铁路新增二线、桂林经河池至百色、柳州经河池至百色等项目前期工作。

推进水利基础设施建设大会战。完成投资16亿元以上。力争开工建设金城江水库、巴马县所略水库扩容工程、凤山县上林水库等3个中小型水库项目，加快推进6个中小型水库项目建设，基本建成5个小型水库，新增库容790万立方米。以特色农业节水灌溉项目为重点，新增恢复及改善灌溉面积15.5万亩。实施中小河流治理项目15个，抗旱引调提水工程项目26个，稳步提升防洪抗旱能力。加快桂西北贫困地区（河池）特色农业节水灌溉项目、桂西北扶贫治旱红水河灌区工程的前期工作。

加强能源基础设施建设。大力实施县县通天然气工程，推进天然气支线管道及县域支线同步配套建设。推动宜州琦泉生物质发电、中电国际宜州燃气热电冷联产等项目开工建设。推进送变电工程项目建设，完成投资8亿元。加快实施新一轮农村电网改造工程，不断完善电网结构，提高农网供电能力和供电可靠性。

精心谋划项目前期工作。结合国家政策、产业导向、资金流向，围绕脱贫攻坚、工业转型升级、特色产业发展、生态养生旅游、交通水利基础设施等领域，安排项目前期经费，谋划实施一批符合新发展理念、事关转型发展、增强发展后劲的重大项目。全市项目库项目滚动保持在350个以上，总投资1000亿元以上。

做好项目建设要素保障。继续实行领导干部联系重大项目责任制，健全完善项目推进协调机制，落实项目建设主体责任，强化绩效考评督查，形成项目推进的强大合力。定期组织开展重大项目建设并审联审活动，精减审批环节，缩短审批时限。创新投融资方式，积极争取金融机构信贷支持，采取政府和社会资本合作（PPP）、总承包（EPC）、政府购买服务等模式，引入社会资本参与各类项目建设。实行征地拆迁属地负责制，明确城区政府为市本级征地拆迁工作的主体，健全住建、土地储备、综合执法等部门参加的征地拆迁指挥部和联席会议制度，确保项目建设顺利推进。

（六）统筹城乡协调发展，营造山清水秀的自然生态。山清水秀生态美是河池的金字招牌。必须坚定生态优先理念，深入实施绿色发展战略，建设独具特色的现代宜居城市，让河池青山常在、绿水常流、空气常新。

健全城乡规划体系。修编完善城乡总体规划及专项规划、控制性详细规划，全面完成河池市总体规划修编、宜州新区一期和高铁新区控制性详细规划、园博园修建性详细规划编制的成果报批工作。继续推进村镇建设、美丽乡村、村庄整治规划编制工作。修编完善土地利用总体规划、第三轮矿产资源总体规划。统筹抓好给排水、燃气、环卫、海绵城市、地下综合管廊等专项规划。

加快城镇建设步伐。有序稳妥推进行政区划调整，加快金宜同城化发展和金城江—宜州城市走廊建设，打造一批特色小镇，积极创建宜居城市。金城江城区要围绕释放发展空间、完善城市功能的思路，加大基础设施改造升级力度，重点推进体育场、辉乐桥、民族公园三期等“十大城建”项目建设，启动连接高铁站市政道路改造工程前期工作，提升城市综合承载功能。宜州新区要启动新区道路交通基础设施和园博园项目建设。积极实施大县城战略，根据资源禀赋、发展基础和功能定位，加强基础设施建设，强化产业、服务和宜居功能，提升品位，全面推进县城提质扩容升级。推进宜州、环江、东兰等3个县（市）新型城镇化示范县建设。结合新型城镇化建设，大力发展县域经济，因县制宜、优势开发、特色发展，促进县域经济水平整体提高。深化户籍制度改革，探索建立居住证与教育、医疗、保障房等基本公共服务供给衔接制度，提高户籍人口城镇化率。

提升城市管理水平。健全完善城市管理长效机制，推动数字城市和智慧城市发展，构建“大城管”新格局。实施“城市畅通工程”，全面加强城市交通管理。加大“两违”整治力度，创建无违建社区（村屯）、无违建街道（乡镇）。巩固提升自治区卫生城市创建成果，开展环境卫生综合整治活动，实现城市主次道路保洁全覆盖。广泛开展城市文明教育，不断提升城市形象。

推进“美丽河池”乡村建设。巩固提升清洁乡村、生态乡村建设成果，推进宜居乡村建设，开展产业富民、服务惠民、基础便民三个专项活动，全面改善农村人居环境。推进沿江沿河沿路特色城镇建设，打造红水河和龙江河观光休闲、盘阳河健康养生、刘三姐文化旅游、高速交通网沿线民族风情等名镇名村。着力抓好自治区层面统筹推进的百镇示范和少数民族乡基础建设。全面保护历史文化遗产、自然景观资源、传统村落民居，抓好54个广西乡土特色示范村屯建设。

推进生态文明建设。积极争取国家、自治区支持，加快创建国家生态文明先行示范区。大力开展石漠化治理，深入实施“绿满八桂”造林绿化升级版、新一轮退耕还林、珠江防护林等重点工程，完成植树造林30万亩以上。系统推进水污染防治、水生态保护和最严格水资源管理，实施龙岩滩水库生态环境保护项目，严格保护水源涵养地和饮用水源地生态环境。积极推进医疗危废处理。规范11个城镇污水处理厂运行监管，加快推进32个乡镇污水处理厂项目及配套管网建设。做好大气污染防治工作，严控污染物排放，确保全市大气环境质量达标。扎实推进土壤污染综合防治先行区建设，实施重金属污染治理专项工程。抓好中央环境保护督察组反馈的问题整改落实，认真落实环境保护“党政同责、一岗双责”，源头严防、过程严管、后果严惩，坚决守住生态底线，确保不发生重大环境污染事件。

（七）全面深化改革，提升开放合作水平。坚持把改革贯穿于经济社会发展各领域，扎实推进供给侧结构性改革，主动融入区域产业协作，助推经济社会持续健康发展。

深入推进“三去一降一补”。去产能方面，全面完成自治区下达的化解煤炭过剩产能和淘汰落后产能任务。去库存方面，落实完善房地产去库存的各项政策措施，发挥公积金支持住房消费作用，保持房地产平稳健康发展。去杠杆方面，抓好政府存量债务置换工作，积极应对和妥善处置各类金融风险。降成本方面，全面落实降成本政策措施，为企业进一步减负，降低综合成本。补短板方面，结合“十三五”规划确定的重大工程项目，增强教育卫生等公共服务能力，加强基础设施薄弱环节和生态环境保护。

全面推进重要领域和重点环节改革。推进农业供给侧结构性改革，农村土地承包经营权确权登记颁证率达95%以上，农村承包土地流转面积达50万亩以上。开展农村集体资产交易平台建设试点，推动资源变资产、资产变资金、农民变股民，增加农民收入。深化行政审批制度改革，进一步做好简政放权、放管结合、优化服务改革工作，提高行政审批效率。完成政府部门权力清单和责任清单“两单融合”并向社会公布。推进企业“多证合一、一照一码”试点，继续实施个体工商户“两证整合”，支持个体工商户转型升级为企业。认真落实国资国企改革政策措施，以混合所有制改革为突破口，推动国有企业开放性、市场化重组，确保国有资产保值增值。深化质量安全监管和检验检测体制改革，构建“大质量”工作格局。全面启动国有林场改革，推进林权制度改革，扩大政策性保险覆盖面和林权抵押贷款规模。积极推进城市管理执法体制机制改革，逐步构建权责明晰、服务为民、管理优化、执法规范、安全有序的城市管理体制。

提升区域合作水平。对接国家“一带一路”建设，深化面向大西南地区的开放合作。积极参与珠江—西江经济带建设，以中国—东盟博览会、泛珠会、桂港务实合作、桂台经贸论坛等活动为平台，深化与珠三角、长三角、大西南、湘桂黔渝毗邻地区等经济协作。推动黔桂连片特困地区同步小康合作示范区建设。深化与广东深圳市的交流合作，拓宽帮扶领域，充分利用深圳的资金、人才、技术优势，促进产业集聚和产业链延伸。谋划开展“柳来河”基础设施合作建设，联合培育发展特色产业，推动形成产业互动互联的城市圈。

强化精准招商。认真落实招商引资优惠政策，充分利用河池长寿养生、生态、旅游、有色金属、优质饮用水等优势资源，突出新兴产业、健康旅游、生态农业等重点，加大精准有效招商力度，到重点地区、重点企业招商，引进有成长性、有生命力、技术含量高、环境污染低的企业，做大经济规模，推动产业转型升级。严格执行招商引资项目协调推进工作机制，优化招商环境，促进项目及早落地实施。全年招商引资到位资金增长8%以上，利用外资（全口径）增长8%以上。

（八）推进社会民生改善，营造团结和谐的社会生态。坚持以人民为中心的发展思想，织牢民生安全保障网，提高公共服务均等化水平，全力抓好十大类为民办实事工程项目，让全市人民共享改革发展成果。

优先发展教育。深入推进教育振兴行动七大工程，实施教育综合改革，办人民满意的教育。推进学前教育三年行动计划，新增一批示范性幼儿园，实现学前教育三年毛入学率达85%以上。加大义务教育均衡发展工作力度，金城江区通过国家督导核验，宜州、环江、南丹、天峨、东兰、凤山、大化7个县（市）通过自治区评估验收。全面改善义务教育薄弱学校基本办学条件，新建、改扩建学校170所，义务教育巩固率达92.5%以上。引导普通高中特色化、品牌化、多元化发展，不断提高学生综合素质，高中阶段毛入学率达85%以上。积极发展特殊教育，提高适龄残疾儿童入学率。支持和规范民办教育发展。加快发展高等教育，支持河池学院建设地方应用型高水平大学，加强与区内院校合作，提升巴马长寿养生国际旅游学校办学水平。构建现代职业教育体系，加强县级职业技术学校办学能力建设，以广西现代职业技术学院为龙头、河池市职业教育中心学校为骨干、各中职（中专）学校为支撑，完成组建河池市职业教育集团。

促进就业创业。千方百计稳定就业形势，定期举办就业援助月、春风行动、民营企业招聘周、高校毕业生就业服务月等专项就业服务活动。鼓励大众创业万众创新，落实创业优惠政策，扶持失业人员、农村劳动力、高校毕业生、转业退役军人等各类人员自主创业。对贫困家庭、下岗职工、残疾人等就业困难群体进行免费培训，实现零就业家庭动态清零。城镇新增就业1.3万人，新增农村劳动力转移就业5万人次。

提升社会保障水平。落实全民参保计划，巩固城乡居民基本养老保险全覆盖成果，城乡居民基本养老保险参保率达90%以上。建立完善补充医疗保险制度，完善城乡居民大病保险制度。加快推进农村低保制度与扶贫开发政策有效衔接，逐步实现农村低保标准与扶贫标准 “两线合一”。加快推进河池市社会养老示范中心、老年公寓综合楼等项目建设，探索五保供养对象的分类管理，提高特困人员供养水平。切实保障农村留守老人、留守儿童、留守妇女权益，推进农村关爱服务体系建设。完成岩滩水电站水库移民遗留问题处理规划实施总体验收工作，落实水库移民后期扶持政策，足额发放后期扶持资金。大力推进保障性住房工程，建设各类保障性住房、棚户区改造8000套，分配入住3000套。完成农村危房改造2.2万户。

推进健康河池建设。提高改革后城市和县级公立医院的运行效率，健全分级诊疗制度，实行药品零差率销售，简化城镇医疗保险报销程序。加强与北京阜外医院等国内一流医疗机构交流协作，提高医疗水平，造福老区人民。促进中医药事业发展，提高医疗救助、卫生监督、疾病防控、妇幼保健工作水平。加强引进人才，强化全科医生、家庭医生和乡村医生队伍建设，促进医疗资源向基层和农村流动。加强艾滋病防治工作。继续做好计划生育服务管理，探索建立计划生育特殊家庭保障体系。

构建公共文化服务体系。积极培育和践行社会主义核心价值观，深入开展群众性精神文明创建活动，加强社会公德、职业道德、家庭美德、个人品德教育，在全社会营造崇德向善的浓厚氛围。加快推进城乡文化体育基础设施建设，新建村级公共服务中心139个，村村通乡镇无线广播台站10个。加强文化遗产保护利用，建设非遗传承基地、传习所5个。

加强和创新社会治理。坚持党委领导、政府主导、社会协同、公众参与、法治保障，推进社会治理体系法治化、精细化。建设平安河池、法治河池，全面落实社会治安综合治理领导责任制，加强社会治安防控体系建设，扩大“天网”工程覆盖面，全面提升人民群众安全感。健全群众诉求表达、利益协调和权益保护机制，按照法定途径分类处置信访问题，及时排查预警和调处化解社会矛盾。全面推进社区矫正基层基础建设，提高法律服务和保障水平。健全应急管理体系、防灾减灾体系，提高突发事件防范应对能力。加大地质灾害治理力度，确保人民群众生命财产安全。健全安全生产“党政同责、一岗双责、齐抓共管”工作机制，从严从细落实安全生产监管责任和主体责任，坚决遏制重特大安全事故。加强食品药品安全监管，继续创建自治区食品安全示范城市，确保群众饮食用药安全。认真贯彻落实民族政策，推动各民族和睦相处、和谐发展，让各民族手足相亲、守望相助，指导环江毛南族自治县办好30周年县庆活动。建立完善县、乡、村(社区)宗教工作三级网络。推进基层自治组织建设，做好新一届村（社区）换届选举工作。推动国防建设与经济建设协调发展，扎实开展国防动员、国防教育、国防设施保护、兵员征集和双拥共建等工作，推动军地融合发展，在重大项目、重点工程建设中贯彻军地双向需求，注重发挥驻地军（警）部队在脱贫攻坚、应急处突等方面的积极作用，巩固发展军政军民团结良好局面。

三、加强政府自身建设

牢固树立“四个意识”，特别是核心意识、看齐意识，坚决维护以习近平同志为核心的党中央权威，做到思想上绝对忠诚、组织上自觉服从、行动上坚定紧跟。全面贯彻落实党的十八届六中全会和自治区第十一次党代会精神，按照全面从严治党的要求从严治政，加快政府职能和作风转变，建设创新、法治、为民、高效、清廉政府，共同营造风清气正的政治生态。

继续解放思想。革除不利于发展的思维定势和行为习惯，敢于突破不适应新常态的陈规陋习，汇聚大发展、快发展的强大能量。加快转变思维方式，牢固树立新发展理念，通过放开市场、放开产权、放开政策，突破各种束缚障碍，加快河池发展步伐。转变工作方式，建立合理容错、及时纠错和正常免责机制，解决干部能力不足不能为、动力不足不想为、担当不足不敢为等问题，形成忠诚履责、尽心尽责、勇于担责的干事创业环境。

加强依法行政。全面依法履行政府职能，形成有权必有责、用权必担责、滥权必追责的制度体系。坚持严格规范公正文明执法，全面提高政府工作人员法治思维和依法行政能力。依法行使地方立法权，加快对经济体制改革、政府职能转变、保障和改善民生等重点领域的政府立法。自觉接受人大及其常委会的监督，定期向人大及其常委会报告工作，接受政协民主监督，切实做好人大代表议案、建议和政协委员提案办理工作。

坚持勤政为民。加强政务服务基础设施建设，改善各级政务服务中心服务环境。推行“互联网+政务”，拓展网络问政，扩大公众参与，运用网络新媒体，及时回应社会关切，保障人民群众的知情权、参与权、表达权和监督权。坚持问政于民、问需于民、问计于民，经常深入实际、深入基层、深入群众，多到条件艰苦、情况复杂、矛盾突出的地方解决问题。强化时间观念和效率意识，弘扬“立即行动、马上就办、办就办好”的工作作风，简化工作程序，优化工作流程，减少工作环节，提高决策效率，提升办事速度，推动各项工作高效运转。

强化作风和廉政建设。巩固“两学一做”学习教育成果，坚持查纠“四风”不放松，推进作风建设规范化、常态化、长效化。严格落实中央八项规定精神和国务院“约法三章”。坚持经济工作和廉政建设两手抓、两促进，严格履行 “一岗双责”，把政府系统党风廉政建设和反腐败工作推向深入。坚持以公开为常态，不公开为例外，推进行政决策公开、执行公开、管理公开、服务公开、结果公开，让权力在阳光下运行。加强行政监察，推进审计监督，加强重点领域特别是对财政扶贫资金、环保政策和保障性安居工程等落实情况的监察审计，坚决纠正侵害群众利益的不正之风。

各位代表，新的一年充满新的希望，新的征程承载新的梦想。让我们紧密团结在以习近平同志为核心的党中央周围，在自治区党委、政府和市委的坚强领导下，不忘初心，继续前进，真抓实干，开拓进取，为坚决打赢脱贫攻坚战、同步全面建成小康社会奠定坚实基础，以优异成绩迎接党的十九大胜利召开！
